# Out-of-Hospital Cardiac Arrest Patients: Different Donor Pathways for an Existing Donor Pool Still Underestimated—Perspective

**DOI:** 10.3390/jcm14196946

**Published:** 2025-09-30

**Authors:** Chiara Lazzeri, Antonello Grippo, Giuseppe Feltrin, Adriano Peris, Rocco Quatrale

**Affiliations:** 1 Tuscany Regional Transplant Center, Largo Brambilla 3, 50134 Florence, Italy; adriano.peris@gmail.com; 2 SODc Neurofisiopatologia, Dipartimento Neuromuscolo-Scheletrico e Degli Organi di Senso, AOU Careggi, 50134 Firenze, Italy; grippoa@aou-careggi.toscana.it; 3 Italian National Transplant Center, 00162 Rome, Italy; giuseppe.feltrin@iss.it; 4 Dipartimento Di Scienze Neurologiche, UOC Di Neurologia, Ospedale Dell’Angelo AULSS 3 Serenissima, 30174 Venice Mestre, Italy; rocco.quatrale@aussl3.veneto.it

**Keywords:** out-of-hospital cardiac arrest, uncontrolled donation after circulatory arrest, controlled donation after circulatory arrest, neuroprognostication, health care system

## Abstract

The clinical pathway of a patient who experiences cardiac arrest and subsequently dies (with or without organ donation) is complex. It involves uncontrolled (u-) donation after circulatory death (DCD), controlled (c-) DCD, and donor after brain death (DBD). The present paper aims to summarize existing evidence on organ donation rates among out-of-hospital cardiac arrest (OHCA) patients, with a focus on these three donor categories (uDCD, DBD, and cDCD). Furthermore, the potential to expand each donor pathway in OHCA patients will be highlighted, based on available evidence. Among non-survivor OHCA patients, the prevalence of brain death (BD) is estimated to be low, though reported data are not uniform. The diagnosis of BD is made 3 to 6 days after return of spontaneous circulation. The implementation of uDCD is known to be quite challenging due to logistical, ethical, and resource issues. Its rationale is still well grounded, mainly considering two factors: (a) the high incidence of OHCA, such that uDCD donors can be considered an existing pool of potential donors; (b) the uDCD pathway shows feasibility both under organizational (i.e., only lung uDCD program) and clinical views (normothermic regional perfusion, ex vivo machine perfusion, and an appropriate donor–recipient match). Controlled DCDs are donors who died after a planned withdrawal of life-sustaining therapy (WLST). Data on the percentage of cDCD among OHCA patients is not uniform since the percentage of utilized cDCD has been estimated at around 10%. According to available evidence, each donor pathway in OHCA has the potential to be expanded, mainly by the identification of potential donors and the implementation of DCD programs.

## 1. Introduction

Worldwide, less than 10% of the global need for solid organ transplants is met. Consequently, there is a persistent mismatch between the availability of organs for transplantation and the demand [1].

In 2023, the International Liaison Committee on Resuscitation (ILCOR) published a position paper defining the potential for organ donation among patients experiencing out-of-hospital cardiac arrest (OHCA). The document also provided a rationale for including organ donation as a key clinical outcome in all future cardiac arrest clinical trials and registries.

OHCA remains a major public health issue, with an incidence rate of between 30 and 60 cases per 100,000 people per year worldwide. According to the 2021 American Heart Association (AHA) Statistical Update, the survival rate following OHCA is only 5–10%. In contrast to other cardiovascular diseases, such as acute myocardial infarction and stroke, where mortality rates have decreased in recent decades, there has been little improvement in survival rates following time-sensitive cardiac arrest treatment over the past 30 years [2,3,4]. The EuReCa-THREE study is an international prospective cohort study that was conducted across 28 countries between September and November 2022. It reported a return of spontaneous circulation (ROSC) rate of 31.2% (range, 17.0–42.7) after OHCA [5], with geographic variation [6]. However, data from Italy are limited. A recent meta-analysis including 42 studies from 13 of 20 Italian regions found an overall incidence of OHCA of 86 per 100,000 population per year (range, 10–190). Survival at the longest available follow-up was 9.0% (95% CI, 6.7–12%; 30 studies, 15,195 patients) [7]. Similar results emerge from a recent meta-analysis of 141 studies, which reported survival to hospital discharge at 8.8% (95% CI, 8.2–9.4%), 1-month survival at 10.7% (95% CI, 9.1–13.3%), and 1-year survival at 7.7% (95% CI, 5.8–9.5%) [8].

The clinical pathway from cardiac arrest to death (with or without organ donation) is complex [9]. The uncontrolled donation after circulatory death (uDCD) pathway may be implemented for patients with refractory cardiac arrest for whom no therapeutic options have been identified by the treating team [10,11,12]. Some OHCA patients who achieve ROSC may progress to brain death and become donors after brain death (DBD), while others may be considered for controlled DCD (cDCD) more recently. A recent International Liaison Committee on Resuscitation (ILCOR) systematic review identified 33 observational studies of organ donation after donor cardiac arrest [13]. No significant differences were observed in graft function or recipient survival across any of the organ grafts studied (heart, lung, kidney, pancreas, liver, and intestine), with organs from donors who died after an initially successful resuscitation compared with those who had not received cardiopulmonary resuscitation (CPR).

Our manuscript has two objectives: (a) summarizing the current evidence on donation rates among OHCA patients, focusing on three different donor categories—uDCD, DBD, and cDCD; (b) highlighting the potential for expanding each pathway based on the available data. Organ shortage should prompt us to implement and/or expand each donor pathway.

Methods: A PubMed search was conducted using the following keywords: cardiac arrest/brain, death/DCD, and controlled/DCD uncontrolled. Only articles written in English were considered; case reports and case series were excluded.

## 2. Donor After Brain Death

Among non-survivor OHCA patients, the prevalence of brain death (BD) is relatively low, although reported data are inconsistent [14,15]. In one study including 162 patients (131 OHCAs and 31 intra-hospital cardiac arrests—IHCAs) treated with targeted temperature management, 86 patients died, among whom BD was the most common cause of death among these patients (58/86, 67%), corresponding to a BD prevalence of 34% [16]. A systematic review of 26 studies involving 23,388 adult patients who had been resuscitated following cardiac arrest (mostly OHCA) identified BD in only 1830 cases (7.8%) [17]. A meta-analysis of 16 studies on conventional CPR and 10 on extracorporeal CPR (ECPR) conducted between 2002 and 2016 reported an overall BD prevalence of 12.6%. More than 40% of BD patients were able to donate organs. Notably, the prevalence of BD among patients resuscitated with ECPR was more than three times higher than among those treated with conventional CPR, likely due to longer arrest times. Similar findings were recently reported in a retrospective study of 307 OHCA patients treated with ECPR in Milan (Italy, 2013–2022), where BD prevalence was 17% [18], while in a small cohort (112 patients) of OHCA patients treated with eCPR, brain death occurred in 22% [19].

BD is usually established three to six days after ROSC [20,21], although the available data are heterogeneous, mainly due to differences in inclusion criteria among study protocols. In a French retrospective study of 214 resuscitated OHCA patients (2005–2015) [22], the median time from ICU admission to BD diagnosis was two days (range, 2–3). By contrast, a larger cohort of 569 OHCA patients who were alive at 24 h after ICU admission [23] demonstrated a mean time to BD diagnosis of 5.3 ± 5.1 days.

Data on organ donation rates among OHCA patients who died according to neurological criteria are not consistent, mainly due to differences in local protocols. In a large cohort of 3061 OHCA patients, BD was diagnosed in 481 patients (16%), 136 of whom became organ donors (28%) [24]. A retrospective cohort study from South Australia (2011–2016) investigated non-traumatic OHCA patients who were included in a hospital-based registry and deceased at hospital discharge [9]. The aim was to compare the clinical characteristics, the timing of WLST and death, and the etiology that precipitated death between OHCA patients who were treated in hospital within a local health network. Of the 213 patients who died, 69 died in the emergency department (mostly from cardiac instability) [75%], with the remaining [25%] dying from WLTS [withdrawal of life-sustaining therapy]); in addition, 144 died during hospitalization. According to the local protocol, the decision to perform WLST was made by a physician in consultation with a substitute decision maker, taking into account the known or perceived wishes of the patient. Modes of in-hospital death included WLST (47%), followed by BD (24%), non-neurological WLST (15%), and cardiovascular instability (14%). Overall, organ donation occurred in 25 patients: 2 (3%) with early ED death and 23 (16%) with late hospital death. Among those who died from BD, 15 (43%) became organ donors. No other data was provided.

According to the available data, the percentage of BD among OHCAs admitted to the ICU is about 10–15%. Further research is needed to assess the incidence of BD among OHCAs in different countries and/or in different regions, specifically to evaluate the percentage of organ donors among BD patients.

## 3. Uncontrolled Donation After Circulatory Death

According to the Global Observatory on Donation and Transplantation [1], uDCD accounted for 1.3% of all DCD activity in 2023 (150 donors), which is slightly higher than in 2022, when it accounted for 1% (106 donors). Controlled DCD represented 93% (10,592 donors). In Italy, data from the National Transplant Center for 2023 show that uDCD activity (limited to the lung program) accounted for 11% of all DCD, increasing to 19% in 2024. Currently, 21 centers in Italy perform both uDCD and cDCD, 43 focus exclusively on cDCD, and 8 are dedicated solely to uDCD.

The main challenge in uDCD is to manage ischemia–reperfusion injuries by preserving the donor organ in a timely manner. France and Spain routinely use normothermic regional perfusion (NRP) to facilitate uDCD donation. In Italy, both NRP and ex vivo perfusion are mandatory due to a legal requirement for a 20 min “no-touch” period before death certification. Kidney transplant outcomes from uDCD are promising [25,26,27,28].

In Europe, uDCD has been introduced with notable success in France [29] and Spain [30]. uDCD programs are also active in Italy [31,32], the Netherlands [33,34], Portugal [35,36], and Poland [37], and they are being developed in Belgium [25,38], Russia [39,40], and Slovenia [41].

The implementation of uDCD is known to be quite challenging due to logistical, ethical, and resource issues [10,42,43,44]. Despite these barriers, however, two factors continue to support the rationale for uDCD implementation:

1. The high incidence and unchanged mortality of OHCA, which leaves a large pool of potential donors with no prospect of survival.
2. The demonstrated feasibility of uDCD programs from organizational (e.g., lung-only programs) and clinical (e.g., NRP, ex vivo perfusion, and donor–recipient matching) perspectives.

### 3.1. Organizational Issues

To date, the uDCD program has two different organizational pathways: (a) the so-called bicompartmental model, which can be implemented in hospitals equipped with an experienced NRP team, available 24 h/7 d; (b) the uDCD-only program, which can be implemented even in hospitals without an NRP team.

In both settings, an integrated approach with the emergency system is necessary, and witnessed cardiac arrest is an essential inclusion criterion. Local procurement coordinators are only involved after death certification. Despite time constraints, it is essential to maintain accurate and rigorous donor selection criteria, in which local procurement coordinators play a pivotal role. The transplant system should perform an appropriate and rapid allocation process, mainly based on a highly selective recipient.

### 3.2. Clinical Issues

#### 3.2.1. Bicompartmental Model

In Italy, donor management during NRP is performed by the local coordinator in collaboration with the Regional Center for Transplant Coordination [18,45]. The targets include maintaining NRP flows of more than 2 L/min and correcting acid–base imbalances, electrolyte disturbances, and glucose abnormalities. Blood samples are obtained at the start of the procedure and then again two and four hours afterward to monitor lactate, liver transaminases, and glucose. Donor evaluation and donor risk assessment are performed during this timeframe (from death certification to organ retrieval) by the local transplant coordinator (donor evaluation) and the regional transplant center (donor evaluation and risk assessment) [43,46].

A biopsy is mandatory for evaluating both the liver and kidneys. Suitability assessment is complex and based on multiple parameters: NRP performance (particularly blood flow and serial lactate values), histological findings at biopsy, macroscopic organ appearance, and parameters during ex vivo perfusion [47]. The transplant center’s experience with uDCD donors is crucial for assessing both organs and matching donors–recipients.

#### 3.2.2. Only-Lung uDCD

Lungs from uDCDs [42,48] have been transplanted in several European countries (France, the Netherlands, Spain, and Italy) with promising [42,49,50] results. Existing protocols are not homogeneous, especially regarding lung preservation methods; the definition of warm ischemic time (WIT); and, finally, the use of ex vivo perfusion. The two main lung preservation techniques used for uDCDs are topical cooling (Spanish group) and protective ventilation. Despite these differences in protocols, lungs from uDCD donors show promising results, and the possibility of optimizing ex vivo lung perfusion could further increase their utilization.

Lungs from uDCD may offer some advantages. For example, lungs recovered from uDCD donors do not undergo the injuries observed in brain death donors, such as the upregulation of innate immunity, “cytokine storm”, and the effects of prolonged mechanical ventilation [13,51,52]. Different from cDCD donors, lungs from uDCDs are not injured by prolonged mechanical ventilation and ventilatory-associated pneumonia [53].

Research into the clinical factors that influence outcomes in uDCD donors remains limited. The influence of factors related to CPR on organ outcomes is still under investigation. A retrospective, descriptive, analytical, cross-sectional study based on data from the Regional Registry of Donation and Transplantation (CORE registry) in the Community of Madrid (2007–2017) [54] found a relationship between organ donation and the age and body mass index (BMI) of uDCD donors. uDCD donors aged 45 to 55 with a BMI of 25 to 30 were found to donate organs more frequently. Capnometry values during resuscitation were associated with kidney transplant, with higher values observed in grafted kidneys with respect to kidneys rejected from transplantation [55]. This retrospective study examined 37 uDCDs and 55 transplanted kidneys between 2013 and 2017. Similar correlations were observed in 12 utilized uDCDs from which 22 kidneys were recovered [56]: a correlation was seen between the highest capnometry values and less need for post-transplant dialysis (24 mmHg, *p* < 0.017), fewer dialysis sessions, and fewer days to recover correct renal function (Rho 0.47, *p* < 0.044). A significant inverse correlation was observed between the capnometry values at transfer and 1-month post-transplant creatinine levels (Rho 0.62, *p* < 0.033). No significant differences were observed between the capnometry values at transfer and primary nonfunction (PNF) or warm ischemia time.

## 4. Controlled DCD

Controlled DCD refers to donors who died after the planned withdrawal of life-sustaining therapy (WLST). According to the Global Observatory on Donation and Transplantation, cDCD activity accounts for most of the DCD activity worldwide (more than 90%), with a slight increase observed between 2022 and 2023 (92 vs. 93%) [1]. In Italy, the National Transplant Center reported a 57% increase in cDCD activity in 2024 compared to 2023. However, obtaining high-quality data on cDCD rates among comatose OHCA patients is difficult, as published studies often fail to provide the details of patients’ clinical courses.

Table 1 shows evidence on cDCD activity from comatose OHCA patients. In 2014, a study on cDCDs (United States) [57] reported that among 991 patients admitted between 2005 and 2011 (91% after OHCA), 560 died (57%). Of these, 530 (94.6%) were referred to an organ procurement organization, of whom 389 (73%) were considered potential donors and 75 (13%) were utilized as donors. A retrospective audit of 514 OHCA patients admitted to a UK regional cardiac arrest center ICU found that 273 died. Of these, 106 (39%) were referred to a specialist nurse for organ donation, and 39 (14%) became potential cDCDs [58]. Of these, 9 (3%) were DNDD and 14 (5%) were cDCD; the mechanism of death was not specified for 2 of them. The study could not determine why 61% of patients were not referred to a specialist nurse for organ donation. Another study, performed at the Leeds General Infirmary [59], found that 53 out of 100 comatose OHCA patients admitted to the ICU died. Of these patients, 29 were referred as potential cDCDs, and 7 were utilized as cDCDs. Recent investigations have assessed the potential for cDCD among OHCA patients admitted to ICUs. A large French regional registry in the Greater Paris Area [60] included consecutive comatose OHCA patients who died in the ICU after WLST. The primary endpoint was the potential for organ donation by cDCDs. Of the 4638 OHCA patients admitted to ICUs, 3170 died in the ICU; of these, 1034 died after WLST due to post-anoxic brain injury. According to the French criteria, 421 out of 1034 patients (41%) could have been potential cDCD donors, equating to 55 patients per year in a population of 4.67 million. After standardization for age and sex, the potential for cDCD was found to be 515 patients per year in France (95% CI 471–560). A prospective cohort study of comatose adult OHCA patients at a single academic medical center in the USA between 1 January 2010 and 31 July 2022 found that, of the 2391 patients admitted to the ICU, 325 died after WLST [61]. Interestingly, evidence suggests sex-related differences in the manner of death, with women being more likely than men to undergo early WLST (within 72 h post-ROSC) [62,63,64,65].

Data on cDCD activity among comatose OHCA patients are scarce since few investigations have addressed this topic. Further research is needed to assess the potential cDCD activity among OHCA patients admitted to the ICU.

## 5. Assessment of Brain Injury After OHCA

The assessment of brain injury requires a multimodal approach [66,67], though prognostication in OHCA patients who remain comatose after sedation withdrawal remains highly challenging.

Some studies have investigated the prognostic role of clinical factors in brain death [22,23]. Madeleine et al. [23] identified seven independent predictors of BD in their validation cohort: female sex; non-shockable rhythm; non-cardiac causes of OHCA; neurological causes of OHCA; administration of vasoactive drugs at ICU admission; administration of vasoactive drugs 24 h after ICU admission; and natremia 24 h after admission.

Traditionally, the prognosis of brain injury after cardiac arrest has been categorized using clinical factors such as the location of cardiac arrest, the initial rhythm, and whether the arrest was witnessed. However, this approach has proven to be an inadequate reflection of the full spectrum of disease severity. Given the heterogeneity of patients, there is an increasing focus on identifying early phenotypes of brain injury rather than relying solely on broad clinical categories [68].

Current guidelines recommend assessing most prognostic factors 48 h after return of spontaneous circulation (ROSC), except for neuroimaging findings (e.g., brain computed tomography [CT]) [66]. However, it can be assumed that in cases of severe brain injury, prognostic factors may exhibit changes at an earlier stage, particularly when multiple parameters are considered. Cytotoxic edema can be quantified using head CT to calculate the gray-to-white matter density ratio (GWR) or by qualitatively evaluating sulcal effacement or cisternal compression [69]. A GWR reduction of less than 1.10 within 24 h after cardiac arrest reliably predicts a poor outcome, with an extremely low false-positive rate [70,71,72]. EEG is another non-invasive neuromonitoring tool. Highly malignant EEG patterns, including background suppression, are a sign of severe brain injury. When assessed at 24 h after OHCA, severe somatosensory evoked potential (SEP) [72] findings (bilateral or unilateral absence) have been associated with later development of BD. CT-based GM/WM ratios in the basal ganglia can predict progression toward BD with high specificity, albeit lower sensitivity.

A recent prospective, multicenter study [73] on OHCA survivors from 28 ICUs of the after-ROSC network investigated the performance of the 2021 ERC/ESICM-recommended algorithm for predicting poor outcomes after OHCA. In comatose patients with a Glasgow Coma Scale motor score ≤ 3 at ≥72 h after resuscitation, the following measurements were performed: (1) the accuracy of neurological examination, biomarkers (such as neuron-specific enolase, NSE), electrophysiology (EEG and SSEP), and neuroimaging (brain CT and MRI) for predicting poor outcome (modified Rankin scale score ≥4 at 90 days) and (2) the ability of low or decreasing NSE levels and benign EEG to predict good outcomes in patients whose prognosis remained indeterminate. All comatose OHCA patients who met the ERC-ESICM criteria for poor outcomes after CA had an unfavorable outcome at three months. According to the guidelines [66], a poor outcome is considered likely if two or more of the following predictors are present: (1) pupillary or corneal reflexes are absent at >/= 72 h; (2) bilaterally absent N20 at SEP wave at >/= 24 h; (3) highly malignant EEG (suppressed background or burst suppression) at >/= 24 h; (4) NSE > 60 µg/L at 48 and/or at 72 h; (5) status myoclonus >/= 72 h; or (6) a diffuse and extensive anoxic brain injury or brain CT/MRI. In patients with an indeterminate outcome (approximately half of the study population), favorable signs predicted neurological recovery and reduced prognostic uncertainty. A systematic review of 37 studies on predictors of good neurologic outcome identified the following combination as the most reliable (specificity > 80%; sensitivity, ~40%) [66,74]: (a) neurologic examination: motor response to pain of withdrawal or localization immediately or at 72–96 h after ROSC, (b) normal blood values of neuron-specific enolase (NSE) at 24 h–72 h after ROSC, (c) short-latency somatosensory evoked potential (SSEP) N20 wave amplitude > 4 µV or a continuous background without discharges on electroencephalogram (EEG) within 72 h after ROSC, and d) absent diffusion restriction in the cortex or deep gray matter on MRI on days 2–7 after ROSC.

In cases of devastating brain injury, and whenever continued therapy is deemed futile by treating physicians, withdrawal of life-sustaining therapies (WLST) is performed based on neurological criteria.

A recent international multicenter matched cohort post hoc study [74] assessed the risk of self-fulfilling prophecy in relation to WLST, based on compliance with the current ERC/ESICM criteria for neuroprognostication, in patients who remained unconscious after 72 h post-arrest. Of the 1717 patients included in the study, 497 (29%) underwent WLST for neurological reasons at a median of 143 h (IQR 108–177). Most patients undergoing WLST (303 patients, 61%) retrospectively fulfilled ≥2 ERC/ESICM poor outcome criteria. Notably, none of the patients who met the ≥2 ERC/ESICM criteria achieved a good functional outcome at six months, neither in the WLST cohort nor among the matched controls. These findings suggest that WLST in patients meeting the ≥2 ERC/ESICM unfavorable criteria carries a negligible risk of withdrawing care from patients who might otherwise have achieved good outcomes. In case of indeterminate prognosis, the ERC/ESICM guidelines recommend continued observation and re-evaluation [66]. This “gray zone” may lead to heterogeneous medical evaluations and decisions [64].

## Figures and Tables

**Table 1:** cDCD activity among comatose OHCA patients.

Investigation	Year/Country	Population	cDCD
Reynolds JC et al. (2014) [65]	2005–2011 (USA)	991 OHCA 560 died	75 cDCDs (13%)
Cheetham et al. (2016) [58]	2010–2015 (UK)	514 OHCA 273 died	28 cDCDs (10%)
Tordoff et al. (2016) [59]	2013–2014 (UK)	100 OHCA 53 died	29 potential cDCDs (29%) 7 utilized cDCDs (13%)

## Data Availability

Not applicable.
